# An intensity-based self-supervised domain adaptation method for intervertebral disc segmentation in magnetic resonance imaging

**DOI:** 10.1007/s11548-024-03219-7

**Published:** 2024-07-08

**Authors:** Maria Chiara Fiorentino, Francesca Pia Villani, Rafael Benito Herce, Miguel Angel González Ballester, Adriano Mancini, Karen López-Linares Román

**Affiliations:** 1 Department of Information Engineering, Università Politecnica delle Marche, Ancona, Italy; 2 Department of Humanities, Università degli Studi di Macerata, Macerata, Italy; 3 Digital Health and Biomedical Technologies, Vicomtech Foundation, San Sebastian, Spain; 4 BCN MedTech, Department of Information and Communication Technologies, Universitat Pompeu Fabra, Barcelona, Spain; 5 Institución Catalana de Investigación y Estudios Avanzados (ICREA), Barcelona, Spain; 6 eHealth Group, Bioengineering Area, Biogipuzkoa Health Research Institute, San Sebastian, Spain

**Keywords:** Deep learning, Domain adaptation, Intervertebral disc, Magnetic resonance imaging

## Abstract

**Background and objective:**

Accurate IVD segmentation is crucial for diagnosing and treating spinal conditions. Traditional deep learning methods depend on extensive, annotated datasets, which are hard to acquire. This research proposes an intensity-based self-supervised domain adaptation, using unlabeled multi-domain data to reduce reliance on large annotated datasets.

**Methods:**

The study introduces an innovative method using intensity-based self-supervised learning for IVD segmentation in MRI scans. This approach is particularly suited for IVD segmentations due to its ability to effectively capture the subtle intensity variations that are characteristic of spinal structures. The model, a dual-task system, simultaneously segments IVDs and predicts intensity transformations. This intensity-focused method has the advantages of being easy to train and computationally light, making it highly practical in diverse clinical settings. Trained on unlabeled data from multiple domains, the model learns domain-invariant features, adeptly handling intensity variations across different MRI devices and protocols.

**Results:**

Testing on three public datasets showed that this model outperforms baseline models trained on single-domain data. It handles domain shifts and achieves higher accuracy in IVD segmentation.

**Conclusions:**

This study demonstrates the potential of intensity-based self-supervised domain adaptation for IVD segmentation. It suggests new directions for research in enhancing generalizability across datasets with domain shifts, which can be applied to other medical imaging fields.

**Supplementary Information:**

The online version contains supplementary material available at 10.1007/s11548-024-03219-7.

## Introduction

Intervertebral discs (IVDs) play a key role in spinal flexibility and load distribution [1]. Magnetic resonance imaging (MRI) represents the ideal method for IVD visualization, for its ability to create detailed imaging without harmful ionizing radiation [2]. The precise MRI-based IVD segmentation is critical for diagnosing spinal disorders, guiding treatments, and facilitating accurate spinal interventions [3].

Deep learning (DL) techniques, especially convolutional neural networks (CNNs), have proven to be effective in IVD segmentation [4]. Research mainly uses supervised methods like encoder–decoder architectures [5, 6] and fully convolutional models [7], with growing interest in mixed supervised [8, 9] and semi-supervised approaches [10]. However, these techniques often face challenges with domain shift across different MRI devices and protocols [11], where variability in scanner settings and MR modalities leads to significant image intensity differences [12].

In different fields of medical image analysis, various domain adaptation (DA) techniques have been used to address the challenges associated with transferring knowledge from a source domain to an unlabeled target domain. These techniques include adversarial learning which aligns feature distributions [13], self-ensembling methods that generate pseudo-labels for unlabeled target domain samples using trained model predictions [14], cycle consistency models that synthesize target images from source images [15], and DA methods based on variational autoencoders [16].

**Fig. 1:**
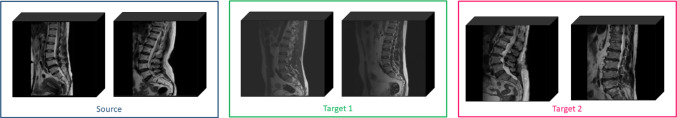
In MRI scans, IVDs display considerable variation in signal intensity, shape, and texture, influenced by aging, degeneration, and pathology, contributing to their diverse appearances. This image highlights the distinct characteristics of the datasets, showing different contrast levels and illuminations. Target 2 in particular, comprises images from different scanners and patients with pathology, resulting in a greater complexity compared to the other datasets

Recently, incorporating auxiliary tasks in computer vision models has shown to be a straightforward way to develop domain-invariant features, improving adaptability across different domains [17]. This approach, particularly beneficial in medical image analysis, simplifies training and addresses generalization challenges posed by data variability across institutions and patient groups. Indeed, various researchers have applied this strategy to solve DA tasks. A structure-driven DA approach for unsupervised cross-modality cardiac segmentation is proposed in [18]. A set of 3D landmarks serves as representative points that embody the heart anatomical structure across various imaging modalities (computed tomography (CT) and MRI). The model learns to predict the positions of the landmarks, facilitating the identification and use of shared structural information. Cardiac structures segmentation from CT and MR volumes is also explored in [19], which uses an edge generation auxiliary task to support the primary segmentation task in the target domain. To cope with domain shift, they employ hierarchical low-level adversarial learning to encourage informative feature suppression hierarchically. Unsupervised DA is explored in [20] for abdominal multi-organ segmentation on CT scans, leveraging the organ location information. A jigsaw puzzle auxiliary task is devised to reconstruct a CT scan from shuffled patches. Additionally, a super-resolution network is used to standardize images from multiple domains. The auxiliary and super-resolution tasks are trained alongside the organ segmentation task to enhance overall performance.

The effectiveness of the DA based on the inclusion of auxiliary tasks strongly depends on the optimal design of the pretext task, which can present challenges, such as the domain shift between the pretext task and the final segmentation domains [21]. Thus, drawing inspiration from recent research in the field of fashion compatibility [22, 23], we recognize the significance of color and texture in analyzing and categorizing visual data. This approach involves the application of color and texture pretext tasks to extract discriminative features while disregarding shape information. Color features in natural images translate into intensity features in medical imaging, as shown in [24], which recently reported a similar approach for histopathological image classification and out-of-distribution detection. This aspect gains importance in MRI, where hardware and software variations result in non-standard tissue intensities, crucial for differentiating IVDs and vertebrae. Therefore, the application of this specific pretext task becomes particularly relevant.

Guided by these considerations, the contributions of this paper can be summarized as follows:

1. First attempt at exploring self-supervised DA for IVD segmentation in MRI.
2. First-ever work that leverages self-supervised learning in medical image segmentation, specifically introducing intensity-pretext tasks for DA. The framework is designed to be end-to-end, offering a straightforward yet effective approach to this complex task.

All the experiments are performed using publicly available datasets to promote comparisons with the presented methods.

## Materials and methods

### Dataset description

We used three MRI datasets: one as the primary source domain (*S*) and two others as target domains (*T*1 and *T*2). They were collected from different medical centers using various MRI scanners, leading to a rich diversity in patient demographics and MRI parameters, essential for enhancing the robustness and applicability of the developed segmentation model.

*S*: The dataset *S*, released by [4], was obtained from a single hospital in China and includes T2-weighted volumetric MRI of 215 subjects, acquired with a 3.0 Tesla MRI scanner (Ingenia, Philips, Amsterdam, Nederlands). For training, validating, and testing the model, the dataset was split into three sets including 172, 19, and 19 volumes, respectively.

*T*1: The dataset *T*1 was released by [25], it consists of T2-weighted MRI from 23 patients, acquired with a 1.5 Tesla MRI scanner of Siemens (Siemens Healthcare, Erlangen, Germany). The dataset was split into three sets for training, validation, and testing, each including 14, 4, and 5 volumes, respectively. The testing set was meticulously labeled by three experienced operators.

*T*2: The dataset *T*2 consists of 30 MR volumes from 29 patients with different medical conditions. Various scanner models from different vendors were used to collect the dataset, including Philips scanners (Achieva, Ingenia, and Elition), Siemens scanners (Avanto, Verio, Espree, Symphony, Amira, Aera, and Magnetom), and a GE scanner (Signa). MR scans from 19 patients were used to train the model, 5 for validation, and 6 for testing. Also in this case, three operators with significant experience carefully assigned labels to the testing set.

For the three datasets, all voxels belonging to IVDs were labeled as 1 while the others were set to 0. For further details on the datasets refer to the corresponding papers. For each dataset, the images included in the test sets were selected to ensure no patient overlap between the train and test sets. Moreover, to guarantee a robust evaluation of our model’s adaptability to a wide range of acquisition contexts, for dataset *T*2, which includes images acquired with various scanner models, the test set was selected to contain images acquired with scanner models not present in the dataset *S*. Samples of the testing set images are shown in Fig. 1.

**Algorithm 1:**
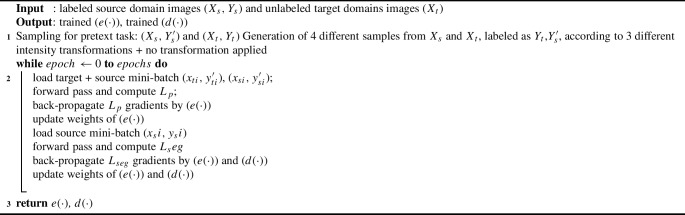
DA method pseudo-code.

### Proposed method

The proposed approach is a dual-task model for IVD segmentation, enhanced by an intensity-based pretext learning task. This additional task generates labels from *S*, *T*1, and *T*2 images based on applied transformations, aiding in feature extraction invariant across domains. The main aim is to improve intensity representation within the embedding space, thereby enhancing the model’s generalization and effectiveness in handling intensity variations in medical images.

**Fig. 2:**
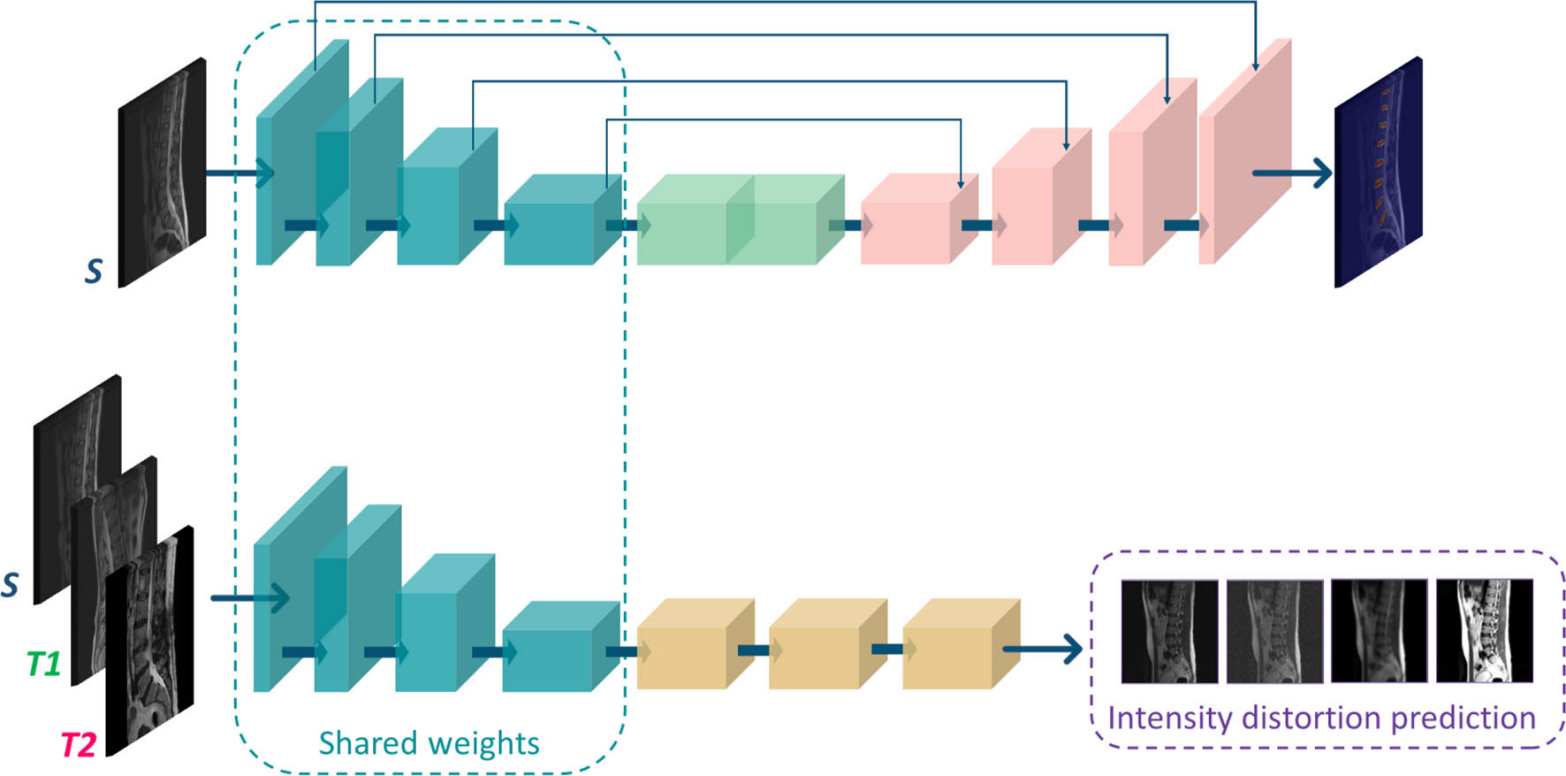
Our proposed framework for self-supervised DA focuses on learning domain-invariant feature intensity representation. This is achieved by incorporating a pretext learning task that automatically generates labels from images of both the source and the two target domains. The pretext task and the main task, which is IVD segmentation, are simultaneously trained using multi-task learning

Figure 2 shows our method focused on IVD semantic segmentation as the main task. We employ an encoder network $e(\cdot )$, which serves as a feature extractor. Additionally, we use a decoder network $d(\cdot )$ to recover spatial information and generate accurate IVD segmentation. This encoder–decoder structure adopts the same architecture as the baseline U-Net described in “Baseline and domain adaptation comparisons”. The $e(\cdot )$ is composed of four blocks, each consisting of two 3D convolutional (conv) layers with a kernel size of $3\times 3\times 3$ and same-padding. Following the conv layers, a rectified linear unit (ReLU) activation function and a batch normalization layer are applied. A max pooling operation with a stride of $2\times 2\times 1$ is performed. At each block, the number of channels doubles, enabling the incremental learning of more complex features. The number of channels starts from 32 and progressively increases to 512. The $e(\cdot )$ also incorporates a bottleneck section which facilitates the connection to the $d(\cdot )$. The bottleneck consists of two additional 3D conv layers with a kernel size of $3\times 3\times 3$ and same-padding. Subsequently, a ReLU activation function and a batch normalization layer are applied to further enhance the learned representations. Similarly to $e(\cdot )$, the $d(\cdot )$ function is composed of four blocks. Each block comprises two 3D conv layers with a kernel size of $3\times 3\times 3$. These layers are followed by a ReLU activation function and an upsampling layer with a kernel size of $2\times 2\times 1$, which reduces the number of feature channels by half. To recover the lost features resulting from downsampling in the $e(\cdot )$ path, the input of each block is concatenated with the corresponding feature maps from $e(\cdot )$. The last block consists of three 3D conv layers, with the first two being followed by a ReLU activation function, and the last one activated by softmax. The number of filters used in the conv layers starts at 256 and is halved in each subsequent block until reaching 32 filters. This CNN is trained end-to-end using labeled samples from the source domain (*S* = {$X_{\textrm{s}}, Y_{\textrm{s}}$}).

To learn intensity invariant features, $e(\cdot )$ is also trained to recognize intensity distortions from both target domains (*T* = {$X_{\textrm{t}}, Y_{\textrm{t}}$}) and source domain ($X_{\textrm{s}}, Y'_{\textrm{s}}$). $Y_{\textrm{t}}$ and $Y'_{\textrm{s}}$ are derived automatically by applying image intensity transformations to their respective images and labeling them based on the specific transformation. This is further detailed in “Pretext tasks” section.

The entire DA method is outlined in Algorithm 1. During forward propagation, samples from both the source and target domains are processed by the shared encoder. Subsequently, the losses for the main task ($L_{\textrm{seg}}$) and the pretext task ($L_{\textrm{p}}$) are calculated, and these losses are then back-propagated and accumulated at $e(\cdot )$. By training $e(\cdot )$ with samples from all three domains, the model learns feature representations that are invariant to domain differences.

### Pretext tasks

The intensity prediction pretext task proposed in this work is inspired by prior works from different fields [17, 22, 23]. Given a set of $N_{\textrm{t}}$ and $N_{\textrm{s}}$ training images from $T = \{x^{\textrm{t}}_{i}\}^{N_{\textrm{t}}}_{i=0}$ and $S = \{x^{\textrm{s}}_{i}\}^{N_{\textrm{s}}}_{i=0}$, respectively, three different sets of intensity transformations, namely Gaussian noise, Gaussian blur, and contrast enhancement are applied. The intensity transformation prediction model $i(\cdot )$, takes the feature maps generated by the function $e(\cdot )$ as input and produces a probability distribution representing different intensity transformations, including the option of no intensity transformation. The $i(\cdot )$ model is composed of three blocks, each containing two 3D conv layers. These conv layers have a kernel size of $3\times 3 \times 3$ and use same-padding. The ReLU activation function is applied after each of them, and batch normalization is performed subsequently. The number of filters used in the conv layers starts at 256 and is halved in each subsequent block until reaching 64 filters. An additional 3D conv layer with a kernel size of $3 \times 3\times 3$, same-padding, and the number of filters denoted as $C=4$ is employed to reduce the number of filters to match the number of classes in the problem. This additional layer is activated by the softmax function. The $L_{\textrm{p}}$ loss is defined as cross-entropy loss.

### Parameter setting

The training process resized images from datasets *S*, *T*1, and *T*2 to $256\times 256 \times 18$ pixels for both the primary task (IVD segmentation) and the pretext task. Optimization was carried out using the Adam optimizer for 100 epochs, with a fixed initial learning rate of 0.001. The primary task used a batch size of 1, while the pretext task used a batch size of 4. Dice loss ($L_{\textrm{seg}}$), effective for class-scarce contexts like IVDs, was the chosen loss function.

To improve generalization, on-the-fly data augmentation was applied during IVD segmentation training. This included geometrical transformations (horizontal flipping, $\pm \,30^{\circ }$ rotation) and intensity transformations (random brightness correction), applied randomly in each training iteration. For the pretext task, only geometrical transformations (random vertical flipping and $\pm \,30^{\circ }$ rotation) were used to enhance perspective generalization without affecting intensity transformation identification.

The best model was selected based on the lowest total loss ($L_{\textrm{total}} = L_{\textrm{seg}} + L_{\textrm{p}}$) in the validation set of *S*. All analyses were conducted using Tensorflow 2.x on an NVIDIA RTX 2080 TI, supported by a Xeon e5 CPU and 128 GB RAM.

**Table 1:** Results of the performance metrics computed on the test sets of the three datasets, obtained from the baseline model (i.e., *U-Net* trained only on the *S* dataset), the proposed model (*t1t2s-int*) and all the other dual-task models trained with various pretext task configurations

Test dataset	Model	DSC (%)	HD (pixels)	Sen (%)	Spec (%)
*S*	*U-Net*	0.86 ± 0.02	28.16 ± 27.69	0.90 ± 0.04	0.99 ± 0.00
	*t1-int*	0.86 ± 0.02	22.32 ± 18.36	0.90 ± 0.04	0.99 ± 0.00
	*t2-int*	0.86 ± 0.02	46.58 ± 84.15	0.90 ± 0.04	0.99 ± 0.00
	*t1t2-int*	0.86 ± 0.02	21.26 ± 18.36	0.90 ± 0.04	0.99 ± 0.00
	*t1t2s-rot*	0.86 ± 0.02	21.25 ± 18.36	0.90 ± 0.04	0.99 ± 0.00
	*t1t2s-rot-int*	0.86 ± 0.02	**18**.**54** ± **17**.**55**	0.90 ± 0.04	0.99 ± 0.00
	*t1t2s-int*	0.86 ± 0.02	21.69 ± 23.68	0.90 ± 0.05	0.99 ± 0.00
*T*1	*U-Net*	0.89 ± 0.03	33.47 ± 37.15	0.88 ± 0.08	0.99 ± 0.00
	*t1-int*	0.91 ± 0.03	24.74 ± 40.92	0.92 ± 0.07	0.99 ± 0.00
	*t2-int*	0.91 ± 0.04	32.82 ± 40.23	0.90 ± 0.07	0.99 ± 0.00
	*t1t2-int*	0.90 ± 0.05	33.20 ± 39.20	0.90 ± 0.08	0.99 ± 0.00
	*t1t2s-rot*	0.88 ± 0.06	27.33 ± 41.72	0.83 ± 0.10	0.99 ± 0.00
	*t1t2s-rot-int*	0.88 ± 0.05	28.76 ± 39.17	0.86 ± 0.10	0.99 ± 0.00
	*t1t2s-int*	**0**.**92** ± **0**.**04**	**13**.**59** ± **17**.**70**	**0**.**90** ± **0**.**07**	0.99 ± 0.00
*T*2	*U-Net*	0.74 ± 0.18	93.59 ± 91.62	0.66 ± 0.22	0.99 ± 0.00
	*t1-int*	0.75 ± 0.18	65.69 ± 70.11	0.67 ± 0.22	0.99 ± 0.00
	*t2-int*	0.76 ± 0.12	85.78 ± 61.42	0.66 ± 0.17	0.99 ± 0.00
	*t1t2-int*	0.73 ± 0.18	56.58 ± 32.11	0.64 ± 0.21	0.99 ± 0.00
	*t1t2s-rot*	0.71 ± 0.10	74.35 ± 57.84	0.58 ± 0.13	0.99 ± 0.00
	*t1t2s-rot-int*	0.75 ± 0.13	71.28 ± 70.67	0.66 ± 0.17	0.99 ± 0.00
	*t1t2s-int*	**0**.**77** ± **0**.**18**	**42**.**83** ± **28**.**11**	**0**.**67** ± **0**.**22**	0.99 ± 0.00

Values are reported as mean ± standard deviation

Significant bold values are best results obtained among the tested models

### Performance metrics

We evaluated the performance of our end-to-end model by calculating metrics for 3D segmentation, as outlined in [26]. Hence, we computed overlap-based metrics on the testing datasets of *S*, *T*1, and *T*2 such as the Dice similarity coefficient (DSC), sensitivity (Sen), and specificity (Spec).

Furthermore, the Hausdorff distance (HD), a distance-based metric, was employed as an additional measure for assessing boundary delineation.

### Baseline and domain adaptation comparisons

We first set the stage by evaluating our proposed strategy against a baseline model, namely the *U-Net* model trained only on the *S* dataset. We then conducted a comprehensive analysis comparing different training data configurations to investigate the impact of introducing different domains into the pretext task:

1. Dual-task model, trained by applying the pretext task exclusively on *T*1 (*t1-int*).
2. Dual-task model, trained by applying the pretext task exclusively on *T*2 (*t2-int*).
3. Dual-task model, trained by applying the pretext task on *T*1 and *T*2 (*t1t2-int*).
4. Dual-task model, trained by applying the pretext task on both *T*1 and *T*2 and on *S* (*t1t2s-int*).

Additionally, we compared the proposed intensity prediction pretext task (*t1t2s-int*) with a traditional rotation prediction task (*t1t2s-rot*). In *t1t2s-rot*, images were randomly rotated (0, 90, 180, or 270$^{\circ }$), and the model, same as in “Pretext tasks” section, was trained to identify the rotation degree. Similar to the previous configuration, the rotation pretext was applied to *T*1, *T*2, and *S* datasets. By comparing these two pretext tasks, we aimed to assess their influence on the dual-task framework and determine the relative effectiveness of intensity prediction against the conventional rotation approach. The combination of the two pretext tasks (*t1t2s-rot-int*) is also explored to estimate how additional tasks impact the result. The same branch from the intensity pretext has been replicated for the rotation pretext.

## Results

**Fig. 3:**
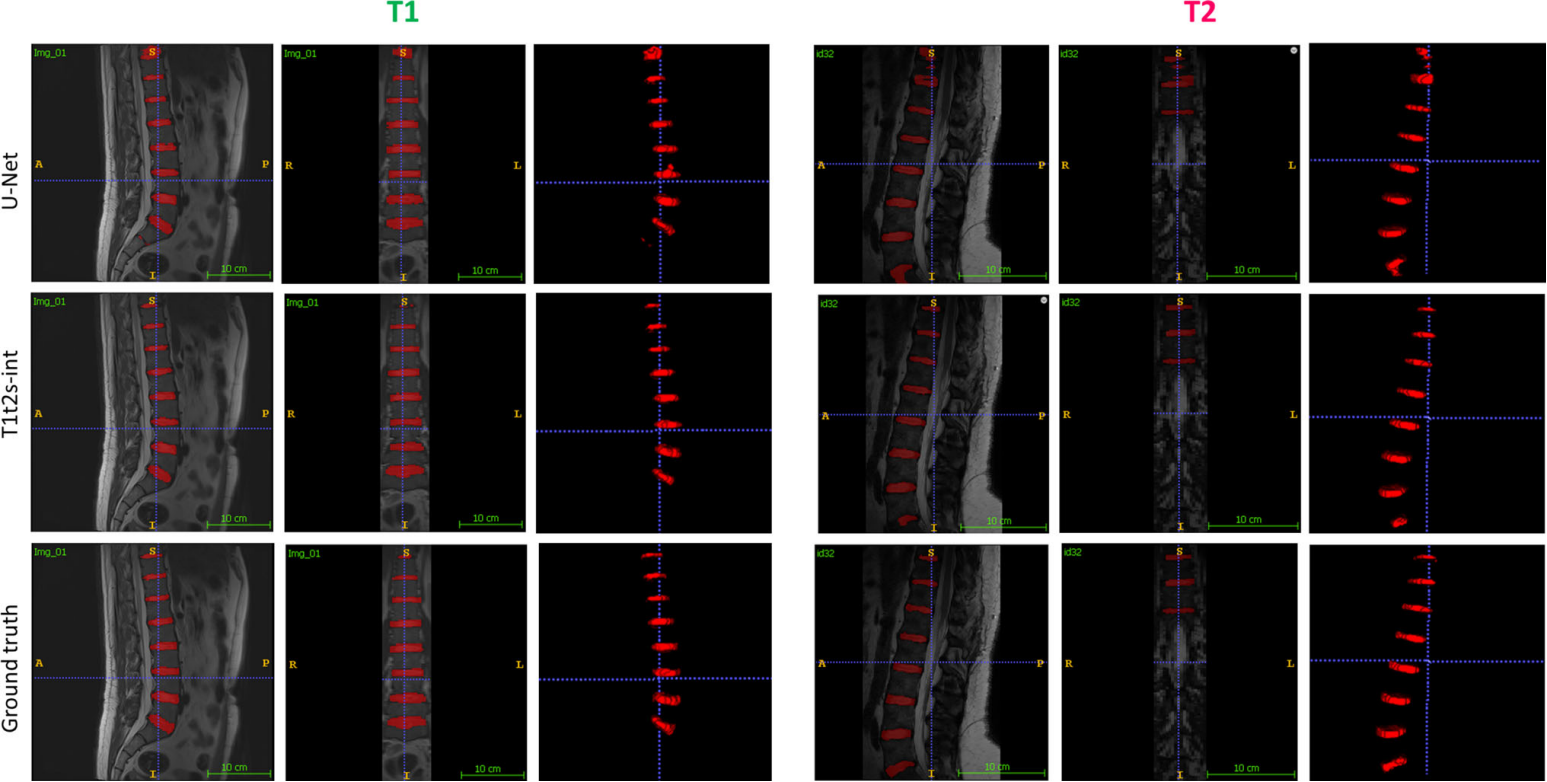
In reviewing the qualitative outcomes of the *U-Net* and *t1t2s-int* training strategies on two randomly selected test images from target 1 and target 2, it is observed that the *U-Net* approach tends to yield less precise segmentation of the discs, particularly those situated in the outer regions of the image

**Fig. 4:**
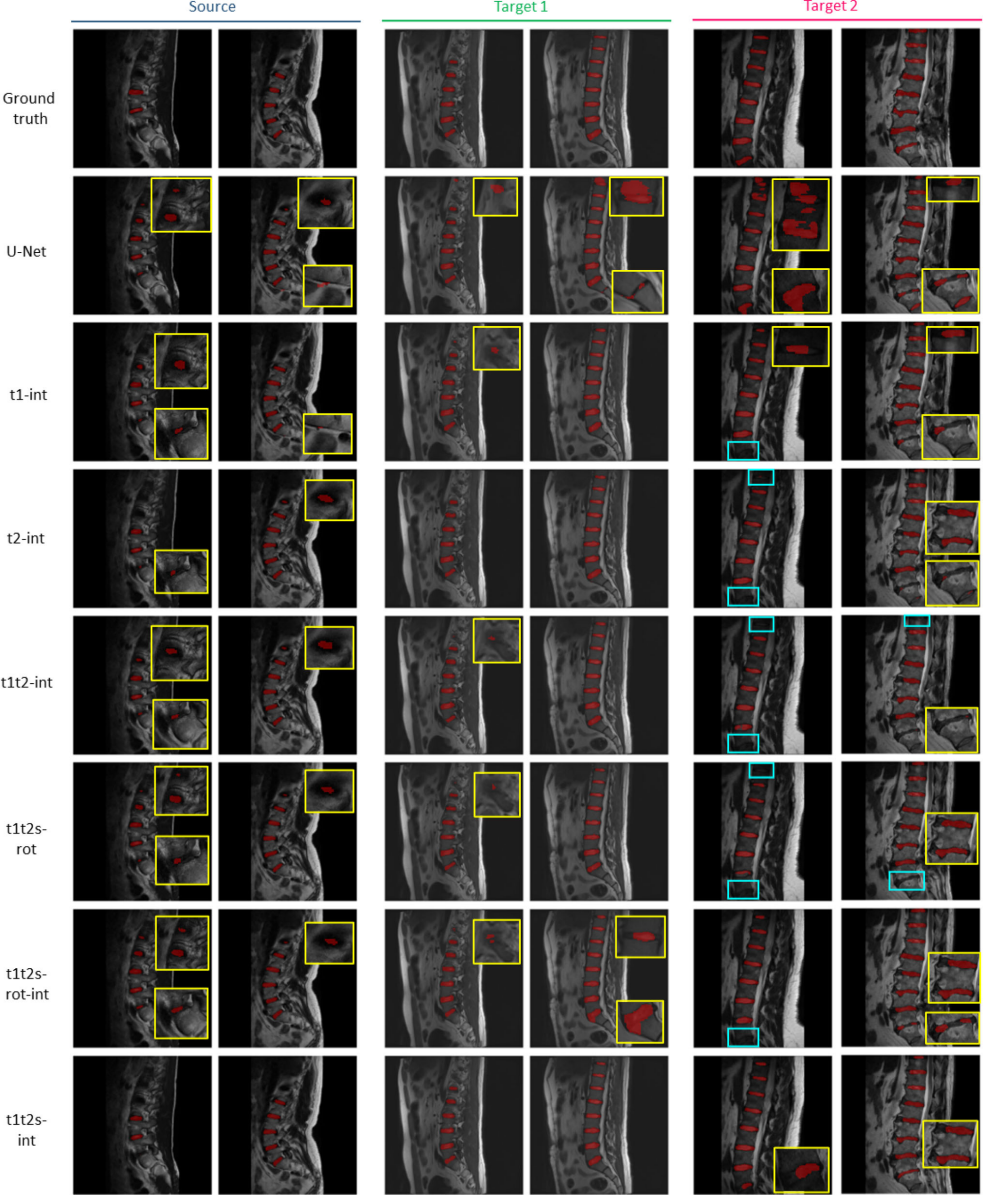
Qualitative results from the comparison of all the tested configurations on two random test images from each of the three datasets (source, target 1, and target 2 from left to right). In the source domain, the improvements brought by the proposed model (*t1t2s-int*) are particularly evident in the accurate segmentation of the contour of the discs. In target 1 and target 2, the proposed model demonstrates fewer false negatives, successfully segmenting all the discs present in the images. Yellow boxes display close-ups of poorly segmented discs, whereas cyan boxes indicate areas of missing segmentation

Results of the performance metrics calculated on the baseline model (i.e., *U-Net*), and on the dual-task models obtained using different training data and pretext tasks (*t1-int*, *t2-int*, *t1t2-int*, *t1t2s-rot*, *t1t2s-rot-int*, and *t1t2s-int*), are presented in Table 1.

For the *S* dataset, all the tested models exhibited comparable performances. The proposed model demonstrated consistency in performance across the different pretext tasks. All training strategies performed well on *T*1, achieving notable peaks in terms of HD, Sen, and Spec when using the *t1t2s-int* configuration. The second best strategy is *t1-int*. Similar trends are observed for *T*2, even though with lower mean values of the metrics compared to *T*1. Also in this case the *t1t2s-int* model achieves the highest DSC and the lowest HD compared to the other tested models. The *t2-int* and *t1t2-int* models obtained slightly higher mean DSC values compared to *t1-int*. When comparing the performances of *t1t2s-int* with a more traditional pretext (*t1t2s-rot*), and with the combination of multiple pretexts (*t1t2s-rot-int*), the former achieved similar results for all metrics in the *S* dataset, for the HD, that was lower in the *t1t2s-rot-int* configuration. The proposed model achieved better results on both target datasets among all metrics. Moreover, in dataset *T*2, introducing only the rotation as pretext task results in a deterioration of performance if compared to *U-Net*. Qualitative results shown in Figs. 3 and 4 further support the effectiveness of the proposed model in accurately segmenting the IVDs while minimizing the presence of segmented spots outside the designated region.

## Discussion

This research explores self-supervised learning for unsupervised DA in IVD segmentation, examining if pretext tasks enhance learning. IVD segmentation in MRI is complicated by their wide appearance variations and limited voxel representation, leading to indistinct boundaries due to the partial volume effect. The study introduces a novel pretext task focusing on predicting intensity variations, such as Gaussian noise, Gaussian blur, and contrast enhancement, which are more pertinent to IVD segmentation than traditional tasks like rotation prediction, aiming to improve model robustness across different imaging sources.

As a result, the model achieves robust segmentation performance across a source and two target datasets containing patients with different pathological conditions and acquired with various scanning devices (namely Philips Ingenia, Siemens, Philips Achiva, Philips Elition GE Sigma).

Evaluating the proposed strategy against the baseline model (*U-Net*), *t1t2s-int* reached the best performance metrics in both *T*1 and *T*2 while maintaining the same performances with respect to *U-Net* in *S*, as shown in Table 1. Figure 3 illustrates the qualitative results of this experiment, showing that the *U-Net* approach tends to produce sub-optimal disc segmentation, especially for discs situated in the outermost regions of the image, highlighting the superiority of the proposed DA model in these challenging regions. This indicates that the strategy not only excelled in the intended domains but also managed to preserve the effectiveness it had demonstrated in the source domain. This aspect is crucial as it ensures that implementing the strategy does not result in any detrimental effects or a decrease in performance in the original domain.

The best training strategy is achieved when incorporating intensity pretext tasks across multiple datasets, including the two target domains and the source domain. This is confirmed by comparing the proposed model with other pretext configurations, as it reached ${\text {DSC}} = 0.92 \pm 0.04$, ${\text {HD}} = 13.59 \pm 17.70$, ${\text {Sen}} = 0.90 \pm 0.07$ for *T*1 and a ${\text {DSC}} = 0.77 \pm 0.18$, ${\text {HD}} = 37.63 \pm 25.40$, ${\text {Sen}} = 0.90 \pm 0.07$ for *T*2. When dealing with pretext applied on only a specific target dataset (*t1-int* and *t2-int*), the performances are better for the dataset on which the pretext was carried out with respect to the other datasets on which the pretext task is not applied, as reported in Table 1. Spec achieved consistently high values of 0.99 in all experiments, underscoring the model’s ability to correctly identify negatives across all cases. A similar trend is observed when applying the pretext task to the two target datasets and not to *S* (*t1t2-int*). This behavior is expected as the model has learned features from just one specific dataset while *t1t2s-int* improves the generalization performance and adaptability across diverse data distributions. This is also evident when qualitatively evaluating the results, as can be observed from Fig. 4. The reduced presence of segmented spots outside the true label region is a critical advancement in IVD segmentation, as it improves the reliability of the segmentation results. This outcome is particularly important in medical applications where precise delineation of the anatomical region of interest (IVDs in this case) is crucial for accurate diagnosis and treatment planning.

Comparing our novel intensity-based pretext task (*t1t2s-int*) with a traditional rotation pretext (*t1t2s-rot*) and with the combination of intensity and rotation pretexts (*t1t2s-rot-int*), we found the intensity-based approach to be more effective, likely because intensities are crucial in differentiating IVDs from vertebral bodies across diverse MRI devices. Furthermore, our findings suggest that introducing a second task may distract the model from focusing on the primary task, resulting in a degradation of performance. This underscores the importance of maintaining task specificity, particularly in domains where nuances in data characteristics, such as MRI intensities, play a critical role.

Our model showed promising results but had limitations. It was tested on a small dataset; using larger datasets could improve its generalizability across populations and imaging protocols. We initially used a basic U-Net for feasibility; future enhancements could include multi-scale pyramid structures and self-attention modules for better performance [27]. Other future developments include the use of a wider variety of intensity-based pretext tasks such as predicting intensity histograms, shapeless local patch discrimination [22], to investigate to enable the model to learn more comprehensive and adaptable features. Additional adversarial loss could also be exploited to further reduce domain shift and improving model generalization [13].

## Conclusions

In our study, we developed an innovative unsupervised domain adaptation method for IVD segmentation, using a dual-task model for segmentation and intensity distortion. Trained on unlabeled multi-domain data, the model learns domain-invariant features, enhancing MRI dataset segmentation. This strategy overcomes intensity variation challenges, outperforming traditional models like U-Net, presenting a promising direction in medical image analysis.

## Supplementary Information

Below is the link to the electronic supplementary material.

Supplementary file 1 (pdf 192 KB)

## References

[1] Liaskos M, Savelonas MA, Asvestas PA, Papageorgiou D, Matsopoulos GK (2021) Vertebrae, IVD and spinal canal boundary extraction on MRI, utilizing CT-trained active shape models. Int J Comput Assist Radiol Surg 16:2201–2214 34643884 10.1007/s11548-021-02502-1

[2] Liaskos M, Savelonas MA, Asvestas PA, Lykissas MG, Matsopoulos GK (2020) Bimodal CT/MRI-based segmentation method for intervertebral disc boundary extraction. Information 11(9):448

[3] Nozawa K, Maki S, Furuya T, Okimatsu S, Inoue T, Yunde A, Miura M, Shiratani Y, Shiga Y, Inage K, Eguchi Y, Ohtori S, Orita S (2023) Magnetic resonance image segmentation of the compressed spinal cord in patients with degenerative cervical myelopathy using convolutional neural networks. Int J Comput Assist Radiol Surg 18(1):45–54 36342593 10.1007/s11548-022-02783-0

[4] Pang S, Pang C, Zhao L, Chen Y, Su Z, Zhou Y, Huang M, Yang W, Lu H, Feng Q (2020) Spineparsenet: spine parsing for volumetric MR image by a two-stage segmentation framework with semantic image representation. IEEE Trans Med Imaging 40(1):262–273 32956047 10.1109/TMI.2020.3025087

[5] Pang S, Pang C, Zhao L, Chen Y, Su Z, Zhou Y, Huang M, Yang W, Lu H, Feng Q (2021) Spineparsenet: spine parsing for volumetric MR image by a two-stage segmentation framework with semantic image representation. IEEE Trans Med Imaging 40(1):262–273 32956047 10.1109/TMI.2020.3025087

[6] Serrador L, Villani FP, Moccia S, Santos CP (2024) Knowledge distillation on individual vertebrae segmentation exploiting 3D U-Net. Comput Med Imaging Graph 113:102350 38340574 10.1016/j.compmedimag.2024.102350

[7] Das P, Pal C, Acharyya A, Chakrabarti A, Basu S (2021) Deep neural network for automated simultaneous intervertebral disc (IVDS) identification and segmentation of multi-modal MR images. Comput Methods Programs Biomed 205:106074 33906011 10.1016/j.cmpb.2021.106074

[8] Pang S, Pang C, Su Z, Lin L, Zhao L, Chen Y, Zhou Y, Lu H, Feng Q (2022) Dgmsnet: spine segmentation for MR image by a detection-guided mixed-supervised segmentation network. Med Image Anal 75:102261 34794095 10.1016/j.media.2021.102261

[9] Kuang X, Cheung JPY, Wong K-YK, Lam WY, Lam CH, Choy RW, Cheng CP, Wu H, Yang C, Wang K, Li Y, Zhang T (2022) Spine-GFlow: a hybrid learning framework for robust multi-tissue segmentation in lumbar MRI without manual annotation. Comput Med Imaging Graph 99:102091 35803034 10.1016/j.compmedimag.2022.102091

[10] Huang M, Zhou S, Chen X, Lai H, Feng Q (2023) Semi-supervised hybrid spine network for segmentation of spine MR images. Comput Med Imaging Graph 107:102245 37245416 10.1016/j.compmedimag.2023.102245

[11] Wang M, Deng W (2018) Deep visual domain adaptation: a survey. Neurocomputing 312:135–153

[12] Neerav K, Krishna C, Christian B, Ender K (2018) A lifelong learning approach to brain MR segmentation across scanners and protocols. In: Medical image computing and computer assisted intervention—MICCAI 2018. Springer, Cham, pp 476–484

[13] Hong J, Yu SC-H, Chen W (2022) Unsupervised domain adaptation for cross-modality liver segmentation via joint adversarial learning and self-learning. App Soft Comput 121:108729

[14] Zhao Z, Zhou F, Xu K, Zeng Z, Guan C, Zhou SK (2023) LE-UDA: label-efficient unsupervised domain adaptation for medical image segmentation. IEEE Trans Med Imaging 42(3):633–646 10.1109/TMI.2022.3214766 36227829

[15] Yang H, Sun J, Carass A, Zhao C, Lee J, Prince JL, Xu Z (2020) Unsupervised MR-to-CT synthesis using structure-constrained cycleGAN. IEEE Trans Med Imaging 39(12):4249–4261 32780700 10.1109/TMI.2020.3015379

[16] He Y, Carass A, Zuo L, Dewey BE, Prince JL (2021) Autoencoder based self-supervised test-time adaptation for medical image analysis. Med Image Anal 72:102136 34246070 10.1016/j.media.2021.102136 PMC8316425

[17] Xu J, Xiao L, López AM (2019) Self-supervised domain adaptation for computer vision tasks. IEEE Access 7:156694–156706

[18] Cui Z, Li C, Du Z, Chen N, Wei G, Chen R, Yang L, Shen D, Wang W (2021) Structure-driven unsupervised domain adaptation for cross-modality cardiac segmentation. IEEE Trans Med Imaging 40(12):3604–3616 10.1109/TMI.2021.3090432 34161240

[19] Xue Y, Feng S, Zhang Y, Zhang X, Wang Y (2020) Dual-task self-supervision for cross-modality domain adaptation. In: Medical image computing and computer assisted intervention—MICCAI 2020. Springer, Cham, pp 408–417

[20] Fu S, Lu Y, Wang Y, Zhou Y, Shen W, Fishman E, Yuille A (2020) Domain adaptive relational reasoning for 3D multi-organ segmentation. In: Medical image computing and computer assisted intervention—MICCAI 2020. Springer, Cham, pp 656–666

[21] Basak H, Yin Z (2023) Pseudo-label guided contrastive learning for semi-supervised medical image segmentation. In: Proceedings of the IEEE/CVF conference on computer vision and pattern recognition, pp 19786–19797

[22] Kim D, Saito K, Mishra S, Sclaroff S, Saenko K, Plummer BA (2021) Self-supervised visual attribute learning for fashion compatibility. In: Proceedings of the IEEE/CVF international conference on computer vision, pp 1057–1066

[23] Xiao L, Yamasaki T (2022) Semi-supervised fashion compatibility prediction by color distortion prediction. arXiv preprint arXiv:2212.14680

[24] Galdran A, Hewitt KJ, Ghaffari Laleh N, Kather JN, Carneiro G, González Ballester MA (2022) Test time transform prediction for open set histopathological image recognition. In: Medical image computing and computer assisted intervention—MICCAI 2022. Springer, Cham, pp 263–272

[25] Chu C, Belavý DL, Armbrecht G, Bansmann M, Felsenberg D, Zheng G (2015) Fully automatic localization and segmentation of 3D vertebral bodies from CT/MR images via a learning-based method. PLoS ONE 10(11):1–22 10.1371/journal.pone.0143327 PMC4658120 26599505

[26] Taha AA, Hanbury A (2015) Metrics for evaluating 3D medical image segmentation: analysis, selection, and tool. BMC Med Imaging 15(1):1–28 26263899 10.1186/s12880-015-0068-x PMC4533825

[27] Du H, Wang J, Liu M, Wang Y, Meijering E (2022) SwinPA-Net: swin transformer-based multiscale feature pyramid aggregation network for medical image segmentation. IEEE Trans Neural Netw Learn Syst 35:5355–5366 10.1109/TNNLS.2022.3204090 36121961

