# Supplementary material for: An intensity-based self-supervised domain adaptation method for intervertebral disc segmentation in magnetic resonance imaging
Source: Int J Comput Assist Radiol Surg. 2024 Jul 8;19(9):1753–61. doi: 10.1007/s11548-024-03219-7 (PMC11365836; doi:10.1007/s11548-024-03219-7)
Supplement: Supplementary file 1 — (pdf 192 KB) [file 11548_2024_3219_MOESM1_ESM.pdf]

An Intensity-based Self-supervised Domain Adaptation Method for Intervertebral Disc Segmentation in Magnetic Resonance Imaging

Maria Chiara Fiorentino*[1], Francesca Pia Villani*[2], Rafael Benito Herce[3], Miguel Angel González Ballester[4,5], Adriano Mancini[1], Karen López-Linares Román[3,6].

1. Department of Information Engineering, Università Politecnica delle Marche, Ancona, Italy
2. Department of Humanities, Università degli Studi di Macerata, Macerata, Italy
3. Digital Health & Biomedical Technologies, Vicomtech Foundation, San Sebastian, Spain
4. BCN MedTech, Department of Information and Communication Technologies, Universitat Pompeu Fabra, Barcelona, Spain
5. Institución Catalana de Investigación y Estudios Avanzados (ICREA), Barcelona, Spain
6. eHealth group, Bioengineering area, Biogipuzkoa Health Research Institute, San Sebastian, Spain

Corresponding author E-mail: f.villani2@unimc.it;
*These authors contributed equally to this work

Supplementary Information

International Journal of Computer Assisted Radiology and Surgery

1 Additional Experiments

This section reports the results of the additional experiments made in this study. The proposed strategy (*t1t2s-int*) was compared with the baseline model (*U-Net* trained only on the source dataset). For this comparison, we conducted a further experiment to investigate the feasibility of segmenting individual intervertebral discs (IVD) within the spine, to capture the differences in the spatial configuration and morphology of each IVD. To achieve this, we employed a principal component analysis (PCA) approach to divide the overall IVD segmentation into separate individual discs segmentation. Fig. 1 and Fig. 2, illustrate the results obtained by both models considering individual IVD segmentation.

In Fig. 1 results for dataset $T1$ are reported, in which *t1t2s-int* shows overall better performances with higher median values and lower interquartile ranges (IQRs) for the Dice similarity coefficient (DSC), sensitivity (*Sen*), and specificity (*Spec*). As regards the Hausdorff Distance (HD), *U-Net* shows higher median values for the discs T10/T11, T12/L1, and L2/L3, with a wider IQR with respect to *t1t2s-int*.

Fig. 2 displays the results for dataset $T2$. The performances are particularly lower with respect to $T1$, but with similar trends: *t1t2s-int* shows better performances than *U-Net* in terms of median values and IQRs for *DSC*, *HD*, and *Sen*. *Spec*, on the other hand, shows comparable median values for the two models for all discs, except for L2/L3, L3/L4, and L5/S.

By examining the performance across individual discs, Fig. 1 clearly demonstrates how the proposed methodology outperforms the others in terms of stability. Specifically, it exhibits consistently favorable results in median and IQR across different IVD on $T1$. Notably, even for the segmentation of challenging IVD such as T10/T11 and L5/S, which are typically more difficult due to their position in the image, the proposed methodology maintains its superior performance. This highlights the robustness of the proposed approach, making it particularly well-suited for accurate and reliable IVD segmentation across different locations within the image. In the case of $T2$ the scenario is slightly different, as the complexity that characterizes this dataset is reflected in the variability of the results between one disc and another. In particular, the discs T10/T11, T12/L1, and L5/S resulted in the lowest *DSC* for both *U-Net* and *t1t2s-int*, even though this latter outperforms the other in all cases. A similar trend can be observed for *HD* and *Sen*.

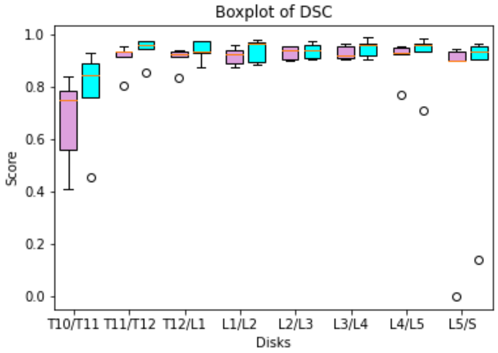

(a)

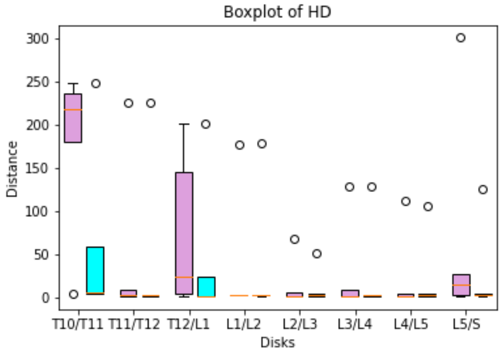

(b)

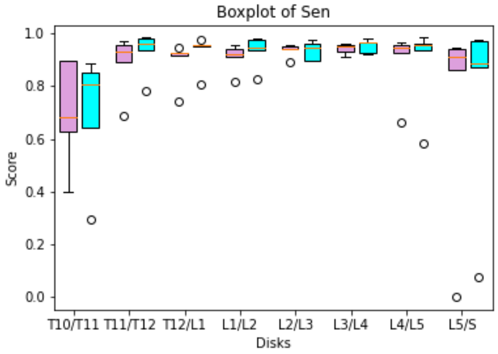

(c)

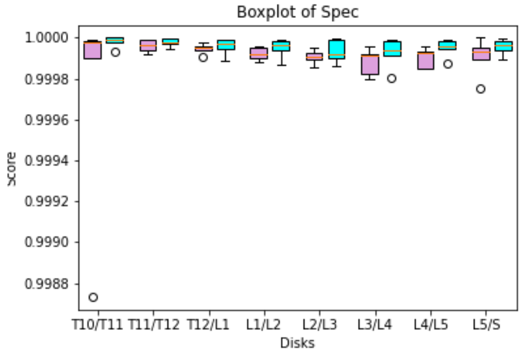

(d)

Figure 1: Boxplots of Dice coefficient (%) (a), Hausdorff distance (pixels) (b), sensitivity (%) (c), and specificity (%) (d) are presented for both *U-Net* (plum) and *t1t2s-int* (cyan) models, specifically for each intervertebral disc in T1. Intervertebral discs are named according to their anatomical position between vertebrae, where T, L, and S denote thoracic, lumbar, and sacral vertebrae respectively.

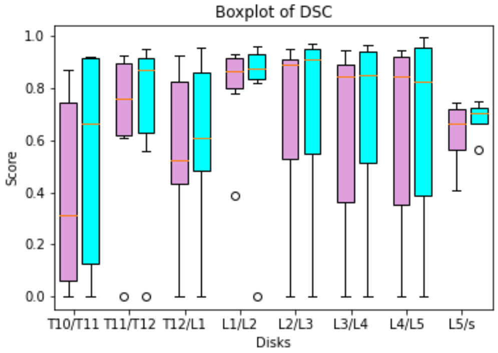

(a)

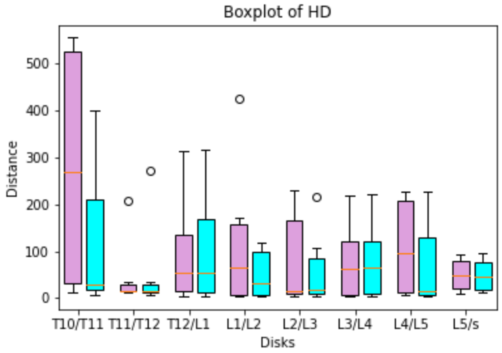

(b)

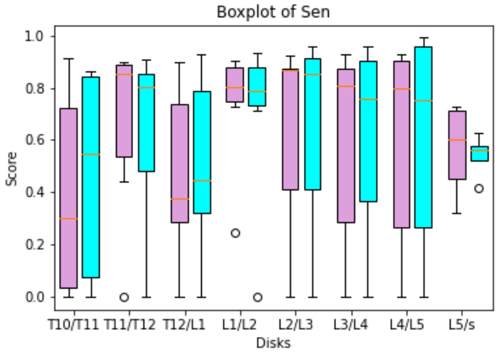

(c)

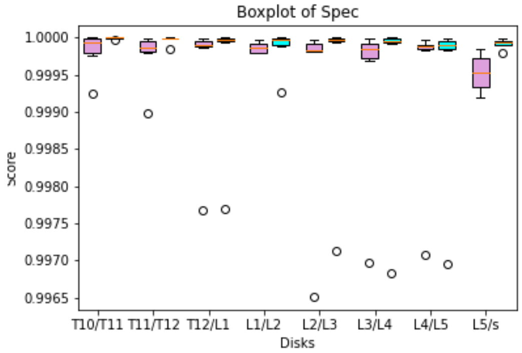

(d)

Figure 2: Boxplots of Dice coefficient (%) (a), Hausdorff distance (pixels) (b), sensitivity (%) (c), and specificity (%) (d) are presented for both *U-Net* (plum) and *t1t2s-int* (cyan) models, specifically for each intervertebral disc in T2. Intervertebral discs are named according to their anatomical position between vertebrae, where T, L, and S denote thoracic, lumbar, and sacral vertebrae respectively.

2 Training curves

In this section, we report and discuss the performance of the model by showing the plots of the loss function (Fig. 3) and the *DSC* metric (Fig. 4), for both training and validation phases over epochs.

As shown in Fig. 3, during the training phase the Dice Loss demonstrates a rapid decline in the initial epochs. The validation loss closely tracks this trend. As the epochs progress, both the training and validation loss values steadily decrease and stabilize, suggesting that the model reaches convergence. On the other hand, Fig. 4 reveals a steady increase in the *DSC* for both training and validation. Throughout the epochs, the *DSC* gradually increases and eventually reaches a plateau, which is indicative of the good segmentation performance of the model.

In both figures, the training and validation curves are close to each other, with no significant divergences, suggesting that the model is not overfitting. The lack of overfitting is further corroborated by the consistent performance in the validation set, which implies that the model generalizes well to unseen data.

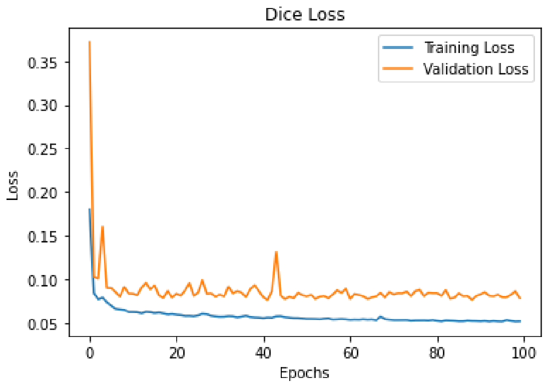

Figure 3: Train and validation loss across epochs. The loss used to train the model is the Dice loss.

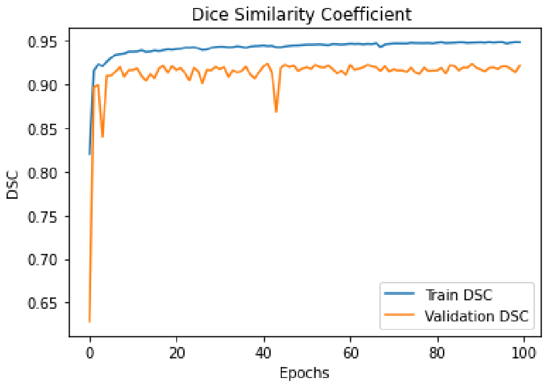

Figure 4: Dice similarity coefficient (DSC) calculated as the metric for the train and validation phases across epochs.
